# The Attitudes of 7–9 Year Old Primary School Students towards Food and Nutrition: Insights from Qualitative FGI Research—The Junior-Edu-Żywienie (JEŻ) Project

**DOI:** 10.3390/nu15224732

**Published:** 2023-11-09

**Authors:** Krystyna Gutkowska, Jadwiga Hamułka, Ewa Czarniecka-Skubina

**Affiliations:** 1 Department of Food Market and Consumer Research, Institute of Human Nutrition Sciences, Warsaw University of Life Sciences (SGGW-WULS), 166 Nowoursynowska Street, 02-787 Warsaw, Poland; krystyna_gutkowska@sggw.edu.pl; 2 Department of Human Nutrition, Institute of Human Nutrition Sciences, Warsaw University of Life Sciences (SGGW-WULS), 166 Nowoursynowska Street, 02-787 Warsaw, Poland; jadwiga_hamulka@sggw.edu.pl; 3 Department of Food Gastronomy and Food Hygiene, Institute of Human Nutrition Sciences, Warsaw University of Life Sciences (SGGW-WULS), 166 Nowoursynowska Street, 02-787 Warsaw, Poland

**Keywords:** primary school students, the age of 7–9 years old, attitudes of primary school students, nutritional knowledge, dietary behaviors, Focus Group Interview (FGI)

## Abstract

Optimal nutrition is one of the most significant environmental factors affecting human health. The aim of this study was to assess the attitudes of primary school students aged 7–9 towards nutrition considering three fundamental components: knowledge, emotional disposition, and dietary behaviors. The research was conducted using the Focus Group Interview (FGI) technique among 78 children. Considering their attitudes towards food and nutrition, four profiles were identified: “engaged”, “obedient”, “reluctant”, and “indifferent”. Children who were “engaged” and “obedient” due to their parents’ involvement in creating their dietary attitudes exhibited the most alignment with the principles of optimal nutrition. Regardless of profile type, it was observed that children were familiar with recommended and unrecommended food products, as well as the role of water in proper nutrition. It was demonstrated that parents wield the most substantial influence on children’s nutrition. As a result, initiatives promoting the proper nutrition and a healthy lifestyle should commence with parents. Children of nutritionally conscious parents tend to eat more healthily and demonstrate a high nutritional awareness. Conversely, the children of busy parents who lack time for meal preparation more frequently replicate their parents’ nutritional mistakes. These findings emphasize the importance of the family environment in shaping the dietary behaviors of children and youth.

## 1. Introduction

Food and proper nutrition are among the most critical environmental factors creating an individual’s health throughout their life. Therefore, they should be not only safe in terms of health quality, but also nutritionally valuable, ensuring both good health and the potential to achieve the highest possible quality of life in emotional and intellectual terms [1,2,3]. Children and youths in their school years deserve special attention in the context of nutrition. This age group is particularly sensitive to the consequences of improper dietary behaviors due to the highly active processes of growth and development during this period, as well as the myriad of external stimuli they are exposed to. Childhood and adolescence constitute a critical period for the formation of dietary habits and behaviors that persist throughout one’s lifetime. The consumption of sweets, fast food, sugary beverages, and the excessive addition of salt and sugar to drinks and dishes can lead to the development of unfavorable dietary behaviors and the emergence of adult-onset conditions, including overweight, obesity, diabetes, and cardiovascular diseases [3,4,5].

The method and quality of nutrition not only influence a child’s physical development, but also have a significant impact on their intellectual, emotional, and social development, as well as their learning ability and school results. This is largely dependent on the efficient functioning of the nervous system, primarily the brain. The brain is arguably the most complex organ in the human body and is considered to be the central command center, influencing every aspect of human life. It controls the functioning of individual organs and systems, regulates all vital functions, and is responsible for processing stimuli that affect our senses, emotions, and behavioral responses. Mental health is the state of functioning in the cognitive, sensory, socio-emotional, behavioral, and motor domains, responsible for realizing an individual’s full potential throughout their life, regardless of the presence or absence of disorders. It depends on the nutritional status resulting from dietary behaviors and is influenced by various factors, as well as knowledge about the significance of supplying specific nutrients [6,7,8,9]. Considering the inadequate nutritional knowledge among the youngest students, it underscores the need for nutrition education and, more broadly, health education. The World Health Organization (WHO) defines health education as a process that enables both individuals and communities to increase their knowledge and awareness of health, ultimately leading to greater control over the determinants of an individual’s and a community’s health. There is no universally applied definition for this term. Wojnarowska [10] defines health education as the process of learning how to optimize care for your own health, as well as the health of others, especially family. The foundation of this process is the acquisition, expansion, and deepening of knowledge about health, including factors that both promote and hinder its maintenance. The essence of this process is the creation of proper attitudes toward health and the cultivation of a pro-health lifestyle.

Lifestyle is defined as the selection of specific behavioral patterns from those available to an individual, which are determined by socio-economic factors and type of motivation [11,12]. Lifestyle, as well as its components, including health-related and dietary behaviors, is created throughout one’s life, starting from early childhood, when the influence of the family is particularly important. During this time, fundamental values are also passed, in accordance with the essence of the family as the primary foundational group. Children imitate their parents’ behaviors, including their dietary and health-related habits, as well as their free time activities [13].

The need for nutritional education stems from various factors, with the primary being the scale of diet-related diseases, which must be unequivocally reversed [5]. This object can only be achieved through a change in attitude toward food and nutrition, especially dietary behaviors. As indicated by Simpson and Freeman [14], in the past three decades, there has been increased utilization of post-positivist and constructivist approaches in the field of health promotion and nutritional education. They emphasize the complexity of health promotion issues. From the perspective of children and youth, they have proposed methodological and theoretical research frameworks that enable researchers to understand health promotion, including from primary school students’ point of view.

Taking into consideration the importance of nutrition education in schools, especially among the youngest primary school students [15,16,17], it is crucial to conduct an assessment that is not only quantitative but also qualitative, diagnosing the attitudes of primary school students towards proper nutrition. Such an assessment will enable the implementation of suitable health promotion activities in schools, appropriate to the students’ perceptions, and the development of appropriate educational programs for them. According to our present knowledge, there is a lack of such research in this age group.

The aim of the study was to analyze the attitudes of primary school students aged 7–9 towards nutrition considering three fundamental components, namely: knowledge, emotional disposition, and dietary behaviors.

## 2. Materials and Methods

### 2.1. Study Design and Participants

Qualitative research was conducted using the Focus Group Interview (FGI) technique in 10 locations in Poland. The locations were diverse in terms of size. The following localities were included in the research: major cities with over 500,000 inhabitants, such as Warsaw; cities with populations ranging from 100,000 to 500,000, including Białystok, Lublin, Kielce, Ostrowiec Świętokrzyski, and Nowy Sącz; small towns with populations of up to 50,000, such as Brańszczyk; and villages of various population sizes ranging from 330 to 2000, including Rosko, Czachówek, and Poręba. Each focus group consisted of from 6 to 8 participants (students), depending on the school’s capacity and parental agreement for the student’s participation in the study. In total, the study involved 78 students aged 7–9 years. The research was conducted as part of the “Junior-Edu-Żywienie” (JEŻ) project.

### 2.2. Moderation of FGI

The Focus Group Interview is a qualitative research technique involving in-depth group discussions, typically with from 6 to 8 participants. Participants are encouraged to express their views on specific topics by a moderator following a predefined moderation script. Participants respond individually to the moderator’s questions, who stimulates them to exchange ideas and actively engage in discussion [14,18,19,20].

The moderation script for the FGI was developed by the authors of the publication and tested in a pilot study. The structure and substantive content are included in Supplement Materials. The study was conducted by two moderators, with an additional third person responsible for recording the interviews and taking notes. The moderator initiated the group discussion with an “ice-breaking” question to help the participants get to know each other, introduce the discussion’s concept (e.g., their thoughts on food, meals, and “healthy” eating), and explain the rules governing group behavior (confidentiality, respect for each other’s opinions, and not interrupting during discussions). The topics covered during the study included children’s approach to nutrition, the perception of their dietary choices, including various food products considered to be “healthy” and “unhealthy”, the consumption of different food groups and beverages, physical activity, and the impact of the COVID-19 pandemic on the dietary and physical activity behaviors of the students. These topics were related to the questions from quantitative surveys conducted by the same team of researchers among primary school students aged 7–9 in a nationwide research sample. The qualitative research was conducted by a specialized firm, the Umbrella Agency Marketing Group. Each in-depth group discussion lasted approximately 90 min.

Written agreement from parents or legal guardians for a child’s participation in the FGI was obtained before each study. The inclusion criteria for the focus group were the students’ age: 7–9 years, the child’s willingness to participate in the study, and the parents/guardians’ consent for their participation in the study. There were no exclusion criteria for participating in the focus groups.

### 2.3. Procedures and Data Analysis

Group discussions were recorded with the participants’ consent in audio format. This fact allowed for the preservation of the participants’ literal statements, which provided researchers with the opportunity to thoroughly familiarize themselves with their content. Interview transcripts and additional notes taken during moderation were transcribed, coded, and preliminarily analyzed by two independent researchers. Subsequently, all connections were examined to reach a consensus. In the analysis of the results, only the name of the locality where the research was conducted was used in reference to the primary school students’ statements.

The material obtained from the in-depth group discussions was subjected to analysis using the principles of grounded theory, with an emphasis on discovering recurring and salient themes resonating during the participants’ discussions. The data analysis was conducted through a seven-step approach: familiarization with the data, thematic coding, identification of sub-themes within the main framework, review and revision of sub-themes, definition and nomenclature of sub-themes, analysis and interpretation of patterns across the entire data set, and the integration of sub-themes into dominant contextual domains.

### 2.4. Ethical Approval

All procedures involving human subjects received the approval of the Ethics Committee of the Institute of Human Nutrition Sciences of the Warsaw University of Life Sciences (No. 18/2022). All of the participants, as well as their parents or legal guardians, provided informed consent to participate in the study. The interview was explained to the participants before the start. The participants could resign from the FGI at any stage without providing a reason.

## 3. Results

### 3.1. The Assessment of Children’s Approach to Nutrition

#### 3.1.1. Perception of the Diet Adopted by the Primary School Students

For the primary school students aged 7–9, it was difficult to definitively determine whether their diet was healthy or not. In most cases, they believed it was “yes” because they brought fruits and vegetables to school, although they were also aware of the occasional consumption of unhealthy meals/products, such as fast food and sweets. The primary school students being surveyed clearly felt uncertain about what constitutes proper nutrition. They were unable to confidently define themselves as “people who follow a healthy diet”. They also did not know what constitutes a proper diet and what deviations are acceptable, such as whether consuming sweets once a week qualifies them as part of those who eat “unhealthily”.

It is also not possible to clearly determine whether the primary school students participating in the study felt they were acting against their own will or if their parents were imposing certain rules and behaviors of “healthy” eating on them. It should be emphasized that the vast majority of students were committed to maintaining a proper way of eating. They made considerable efforts and faced challenges in this direction, such as consuming foods that they may not always enjoy but are considered to be healthy. All the participants clearly stated that food influenced their strength, energy, and health. This is the message they most commonly heard from adults.

According to the primary school students, the main indicators of healthy eating are vegetables and fruits in the diet. The majority of them declared eating these products daily, with a significant portion consuming them in almost every meal. The students of this age paid little attention to the portion size of vegetables and fruits consumed throughout the day. According to them, the presence of vegetables and fruits in the diet is important. The children had a also problem with determining portion sizes. Some of them stated that, for example, consuming one fruit a day (such as an apple, banana, or orange, etc.) is a sufficient amount in their daily diet.

The primary school students could identify products they considered to be indicative of an unhealthy diet. According to them, these are primarily sweets, salty snacks, and fast food products. The primary school students believed that individuals with poor dietary behaviors and a sedentary lifestyle for an extended period of time will, as a consequence, “*become obese, sick, fatigued, generally weak, and, in addition, they will constantly experience sadness and a bad mood*”.

Among the primary school students aged 7–9, sportsmen served as role models for healthy eating. Healthy nutrition is a necessity in their “profession” and passion. Some also mentioned their parents, who regularly engage in activities such as running or going to the gym. Such declarations were more common among students from larger cities and were associated with the lifestyle of individuals in their parents’ age group, typically between 30 and 45 years old.

Sample children statements:

*“Yes, from time to time, I eat healthily, for example, on my birthday, instead of candies, I brought apricots”*

(child, Warsaw)

*“If we eat vegetables or fruits, we can have vitamins that affect everything—our eyes, our bones”*

(child, Kielce)

#### 3.1.2. Typology of Children Participating in the Study

Among the study participants, it is possible to create a typology that distinguishes them based on their level of involvement in issues related to food and nutrition, their awareness of the subject, and selected behavioral aspects in this area. The results allowed for the identification of student profiles as “engaged”, “obedient”, “reluctant”, and “indifferent”. The characteristics of students according to this typology are presented in Table 1.

**“Engaged” primary school students’ profile** 

The “engaged” profile was represented by a small group of surveyed students. They were mainly residents of larger cities and villages located near major urban centers.

Practically with every meal, the students consume vegetables and fruits. Their diets are rich in legumes, fish, whole grain products, and dairy. It can be inferred that the parents of the children representing this profile often discuss topics related to proper nutrition, explaining and answering questions about these issues. In their homes, this is an important, enjoyable, and willingly discussed topic. Nutritional education happens naturally and spontaneously, without compulsion and the use of punishments and prohibitions. It is easy for the children to follow proper habits because they encompass the whole family, and it happens through imitation, further demonstrating the effectiveness of the family environment in creating the attitudes of the youngest toward food and nutrition. A rational way of eating is a choice and lifestyle for them, rather than an obligation. The parents of these students are usually individuals with a high level of nutritional awareness.

Sample statements from children:

*“I just wanted to say that my mother and I make vegetable and fruit chips. And a few times a month, we make homemade pizza, which I like a lot more than the store-bought one”*

(child, Warsaw)

*“I eat the same food as my parents, and they eat very healthily. I like talking to them about food because they explain things so interestingly”*

(child, Ostrowiec Świętokrzyski)

**Primary school students with an “obedient” profile** 

The “obedient” profile was the most numerous group of 7–9-year-old students who participated in the study, including both boys and girls from various locations.

According to representatives of this profile, their parents dedicate a relatively significant amount of time, attention, and energy to dietary principles. They care about and see the importance of implementing proper dietary habits for all family members, especially for their children. The entire family can afford more flexibility and less restrictions during the weekends and shared time for games or watching movies. During these times, they eat homemade popcorn or pizza prepared together. However, during the week, children are closely controlled and monitored in terms of the types and quantities of meals consumed. They only receive “healthy” products for school, which they willingly consume. Students with the “obedient” profile did not show rebellion and rarely expressed opposition to the imposed diet because they were not familiar with any other. The challenge for them lies in gatherings with peers and birthdays, etc., when they discover the world of colorful sweets, snacks, and soft drinks, which seem very appealing and tempting to them.

Sample statements from children:

*“I don’t eat healthy things every day, or I know that when I only eat sweets, I feel bad. Now, my grandma also doesn’t buy me sweet things. I don’t want them, and I don’t need them”*

(child, Rosko)

*“When I ask my father for something sweet, he says that it’s not needed for life at all”*

(child, Rosko)

**“Reluctant” profile students** 

The “reluctant” profile was represented by both girls and boys from various locations. This is a relatively small group, often referred to as “picky eaters”, who have a narrow range of foods they enjoy. As a result, their diet is “fairly healthy” but highly limited and lacking in diversity in terms of nutritional components. They are unwilling to try new foods, making it difficult to expand their diet to include new flavors and types of products. They feel forced to eat and are often described as “pick eaters” or “picky eaters”, which hinders them from trying new foods. This group could be characterized as neophobic. The parents of these students are typically individuals who aim to eat healthily but do not always succeed, or they lead busy lives and do not pay much attention to what they eat, often failing to recognize the problems and risks associated with it.

Sample statements from children:

*“In my opinion, sometimes it’s like we don’t like something because we don’t find it visually appealing. If we don’t see it and eat it, it tastes good”*

(child, Nowy Sącz)

**Primary school students with the “indifferent” profile** 

The profile “indifferent” represents a very small group of students aged 7–9, living in various locations, mainly boys.

Their diet is moderately healthy and rational. They have not developed specific attitudes towards food and nutrition. It can be assumed that the parents of these students ensure certain minimum standards, such as having the child eat lunch at school, bringing a sandwich for a second breakfast, eating fruits and vegetables a few times a week, and limiting sweets without strict restrictions. School is virtually the only place where they may encounter the principles of proper nutrition.

They do not reject valuable and recommended products, but also do not pay much attention to the quantity and frequency of consumption. At the same time, they have a considerable degree of freedom in choosing the products and meals they eat. “Indifferent” students are the least controlled by their parents. Their parents are usually individuals with busy and demanding lifestyles. This is the group that most often, among all students, buys snacks and sweets from vending machines located at schools.

### 3.2. Food and Emotions

Children, asked whether they like to eat, stated that it depends on their level of hunger and the type of meal, as well as their emotional state. Their statements confirm that emotional states influence the consumption of various food products (Table 2).

Sample statements from children:

*“It depends on whether I am sad or happy. When I’m happy, I eat, and when I’m sad, I don’t feel like eating because I feel like I have a full stomach”*

(child, Lublin)

### 3.3. Children’s Eating Style

#### The Number and Type of Meals Consumed throughout the Day by Primary School Students

The vast majority of the surveyed students reported consuming an average of 3–6 meals, but they could not specify how many meals should be eaten in a day. This is due, in part, to the fact that they usually eat at times determined by their parents or school, depending on their location. These students rarely eat on their own when they feel hungry. This is mainly because they have inherited such behaviors from home and kindergarten. Additionally, they are under the constant supervision of teachers, guardians at the after-school program, parents, and grandparents, who dictate the rhythm of their day and meal times.

BREAKFAST

The vast majority of primary school students are aware that it is essential to have breakfast every day because it is necessary for proper functioning throughout the day. It serves as a source of energy for a good start, often referred as “fuel for the day”. Therefore, breakfast is considered as the most important meal of the day. Among younger students, however, having breakfast can be a subject to discussion. A significant portion leave home before having a meal. However, some students, often from larger cities and those raised by single parents, claim that they eat their first meal at school, as a second breakfast, while others do not have breakfast at all. It may seem that a certain group of students at this age often leave home without eating and also do not compensate for this lack of a morning meal at school, which is a serious nutritional mistake affecting their cognitive performance during lessons, related to brain nutrition.

More than likely, more students in lower primary school grades would choose not to eat breakfast if it were not for parental pressure. The research participants expressed that they dislike the morning rush and are often not hungry, so they eat “forcibly”. It seems that if the child could, they would leave the house with an empty stomach, but parents ask them to “eat something”. Often, even half a sandwich or colorful, sweet cereals, which are not allowed during the day, are enough, but parents are willing to change their rules when it comes to breakfast. Table 3 presents the reasons provided by students for not eating breakfast.

Sample statements from children:

*“I only sometimes have breakfast at home. Sometimes, I just have little time, and sometimes I just don’t want to, and I’m not hungry in the morning, or I’ve had enough of something”*

(child, Warsaw)

*“Breakfast gives an energy for the whole day. Dinner and supper are just additions. That’s why breakfast is the most important because it gives signals to the body that it’s time go to work”*

(child, Kielce)

*“Breakfast is the most important. It should last you the entire day, giving you energy and strength. If you do not eat breakfast, you feel so tired and can’t focus on studying or anything else”*

(child, Nowy Sącz)

*“If we do not eat breakfast and go to work or school, we’ll be so tired, and we won’t feel like doing anything”*

(child, Brańszczyk)

*“If someone has time, they eat at home, but if someone sleeps and does not have time, eat at school”*

(child, Poręba)

SECOND BREAKFAST

Second breakfast is a meal consumed by the vast majority of students participating in the study. Only a few individuals admitted to skipping this meal, even though they had it prepared in their school backpacks. It seems that social influence plays a significant role in this matter. Children observe others eating. This fact stimulates their appetite. They also consume their second breakfast because they do not want to be excluded from the group.

Most of the primary school students, for their second breakfast, typically receive sandwiches, sausages, sweet or savory cheese snacks, and fruits. From the students’ statements, it can be concluded that parents would like their children eat at least a snack at school. Therefore, lunchboxes often contain energy bars and other sweets favored by children to supplement their energy levels and, in their perception, enhance concentration.

Primary school students are at an age where they are under the almost constant supervision of their teachers, who signal when it is time to eat second breakfast. In many schools, students eat this meal together with their teachers. The students do this willingly.

*“My mother sometimes packs me an apple, grapes. Sometimes, she even includes a donut. Some cookies too”*

(child, Brańszczyk)

*“Grapes, an energy bar, cheese. I’m sure I’ll get something I like”*

(child, Czachówek)

DINNER

The majority of primary school students participating in the research have their dinner at school. Students whose parents or guardians stay at home full-time typically have dinner at home after classes. This takes place mainly in smaller towns. Students agree that dinner is a very important meal during the day (for some of them, even the most important) because it is substantial, often a two-course meal, nutritious, and well-balanced. It typically consists of meat, potatoes/grains, and vegetables served warm in the middle of the day, providing energy for the rest afternoon.

The decision to purchase dinner at school is made by parents, who consider how many hours their child will be away from home and at what time they will be picked up from school. Unfortunately, children often decide not to consume this meal in its entirety or in part if they dislike dishes served in this meal.

The main factor of having dinner at school is the company of children in the same age. However, most students aged 7–9, regardless of their place of living, prefer to have dinner at home. Children provide the following reasons:

- the meals are prepared at home, not by a catering company;
- taste better, are prepared in a known and preferred way, with trusted and high-quality ingredients;
- there is no need to hurry, there are no time limits;
- the meals are presented more aesthetically and have an appetizing appearance;
- it is possible to consume a larger portion and ask for seconds;
- there is a nicer atmosphere at home, eating in silence, with family;
- better eating conditions, at the table, no one is pushing or rushing;
- it is possible to engage in other activities, such as watching TV;
- the timing is not strictly defined; the family adjusts the mealtime to the child’s hunger, while at school, children must eat at a certain time, regardless of hunger.

Individuals eat two dinners during the day, one at school and the other at home with their family. The latter meal typically occurs at dinnertime and often replaces that particular meal (supper). However, some students, have dinner late. 

For most primary school students, dinner is associated with a family atmosphere and time spent together. It is more commonly enjoyed on weekends, but a large number of students also have dinner with their families during the week. This depends on the daily routine and the work schedule of all family members, especially parents. The students’ place of living does not have an impact on this behavior. Some children have dinner alone in their room or in front of the television or laptop, even when other family members are at home. This situation occurs more frequently in larger cities and in the case of only children.

Sample statements from children:

*“I like to eat while watching TV because it’s like you’re watching and having your meal. When you’re not watching and not eating, it’s a bit boring”*

(child, Kielce)

*“In the first grade, I used to have dinners at the school but I didn’t want to eat anymore because sometimes dinner was during class, even when I was not hungry. At home, there is dinner and I can serve myself at any time”*

(child, Kielce)

*“At home it is more calm, there’s more time, and you can eat more. In school when I finish breakfast, I can not have anything else”*

(child, Rosko)

*“At home, I used to eating what I like”*

(child, Lublin)

*“I do not like the school’s dinners because of different recipes. I’m used to some things, and not to others”*

(child, Białystok)

AFTERNOON SNACK

Among primary school students aged 7–9, an afternoon snack is often described more as a small snack. It is frequently skipped due to a lack of hunger and is sometimes replaced with sweets (energy bar, waffle, and chocolate), and is then referred to as a dessert.

SUPPER

Sometimes, supper is perceived as an unnecessary meal. Children do not feel hungry in the evening, especially after a too substantial dinner combined with a large amount of snacks. There is a general belief that supper should be light. Otherwise, it may cause stomachache.

Sample statements from children:

*“Supper is not very important because when we eat supper and go to sleep and wake up, we’re hungry”*

(child, Brańszczyk)

*“I do not have dinner. When I come home, my second meal is as a late dinner, so I do not eat anything for supper”*

(child, Warsaw)

*“I do not have supper because it is not healthy to eat at night. I used to have supper but then a stomachache so I stopped eating supper. My stomach still hurts, but less. I think it helped me”*

(child, Kielce)

### 3.4. Primary School Students’ Knowledge and Perception of Various Food Product Groups

#### 3.4.1. Knowledge of Foods Considered as ‘Healthy’ and Their Consumption Frequency among Primary School Students

The majority of students consume a variety of products from different food groups, including dairy products, grains, fruits and vegetables, and nuts, as well as pumpkin and sunflower seeds, but with a varying frequency and limited diversity within each group (Table 4). In each discussed food group, the respondents pointed out both their favorite and least favorite products, which they rarely or never consume. Encouraging them to try and diversify their diet with these products is usually challenging in such cases.

During the study, only a few students were able to identify the specific components supplied by certain food product groups. Most of them simply attributed these products to a high content of vitamins and a general positive impact on health and well-being. The term “healthy fats” and dietary fiber posed the most difficulty for them. While these concepts were familiar to the students, they could not attribute specific functions and roles to them. It is clear that the students were unable to determine why certain products are essential in their diet and why they should consume them. Building this awareness from an early age and emphasizing this aspect in school education is important. It will help them to understand why it is crucial to consume products rich in these components and not just perceive them as something their parents force them to eat.

The majority of primary school students were not familiar with the guideline that recommends consuming a minimum of five servings of fruits and vegetables daily. In their opinion, only one serving is sufficient. However, it should be emphasized that the students were not entirely clear on how to interpret the term “serving” (whole fruit or just a piece of it, like a slice of an orange). This was too abstract for them and required clarification. The question about the proportion of vegetables and fruits on a plate yielded diverse responses. Many students answered that it should be half the plate, while a significant number of respondents chose the option of one quarter. However, it is essential to note that, the first option, regardless of gender, place of residence, and the represented approach profile to nutrition, seemed too large, even impossible to eat for most students.

Sample children statements:

*“I really like eating fruits, less vegetables”* (child, Poręba).

*“I hate fish. They have a strange taste and bones. They are also slimy and fatty”* (child, Czachówek).

*“I feel like they swim in my stomach afterwards”* (child, Warsaw).

*“Fish smell, and you can see their eyes”* (child, Czachówek).

#### 3.4.2. Knowledge of Products Considered as “Unhealthy” and the Frequency, Circumstances and Motivations for Consumption

The students had no difficulty identifying products that should not be present in their diets. They expressed awareness that consuming these items could harm their health, potentially lead to overweight issues, and generally result in poor feelings. They also expressed the following opinions:

- Sweet drinks and sweets are responsible for delivering a significant amount of sugar to the body, which can lead to obesity and tooth decay.
- Harmful ingredients such as food coloring, preservatives, and unhealthy chemicals in a product’s composition were noted. The students were familiar with the concept of high-fructose corn syrup, which they believed should be avoided. According to them, good sweets are those without sugar.
- Fast food items were considered to be low in nutrition and providing energy for a short duration. They were perceived as having numerous preservatives and fats.
- Salty snacks were seen as containing salt, which stimulates appetite and retains water in the body.

The study results allow us to identify four main contexts for consuming products not recommended for consumption: family, social, individual, and travel-related (Table 5).

The presented contexts of consuming products that are not recommended for consumption can be divided into controlled and uncontrolled by adults, such as parents, guardians, grandparents, and teachers. In the case of students at this age, the controlled contexts are more common, because they rarely eat meals on their own without supervision. They do not purchase food independently from stores, except for the school store and vending machine at school. When they choose unhealthy products, they usually get them from their family’s supply. Only a small number of students (children) admitted to purchasing sweet sodas, candies, and salty snacks on their own, without their parents’ consent, and sometimes even covertly.

In the case of primary school students aged 7–9, the discussion of fast food primarily arises in the context of family outings and trips, which are supervised by parents or teachers. Children in this age group do not make independent purchases of such items. For young residents of small towns and villages, this issue is almost non-existent, because there is no fast food available in their immediate surroundings. These products are primarily encountered during family trips.

Sample statements from children:

*“Salt/sugar is white death”*

(child, Białystok)

*“They fill you up for a moment, and then your stomach hurts”*

(child, Czachówek)

### 3.5. Students’ Liquid Consumption

Primary school students aged 7–9 belong to a generation that has been instilled with the importance of staying hydrated from a very young age. They most often choose water, which not only quenches their thirst but is also a healthy choice, ensuring the proper functioning of the human body. The majority of students have water bottles and make use of tap water or water fountains on the school premises. In their perception, they consume a significant amount, approximately 1–1.5 L per day, although it should be noted that they may not be fully aware units of measurement and probably drink less. The students have a preference for black or fruit tea (mainly during meals) and homemade compotes and juices (often received from their grandparents).

Respondents occasionally enjoy industrially prepared fruit juices from cartons, typically at home during family meetings, holidays, and special occasions. Parents tend to discourage excessive consumption due to the high sugar content. Some participants also distinguish between fruit juice and fruit-flavored beverages, such as orange drinks. On the other hand, sweetened carbonated beverages are considered to be more of a treat and a rarity than a source of hydration. They are consumed occasionally and mainly under parental supervision.

Sample statements from children:

*“I have this health book that contains information about good and healthy food. It recommends drinking ten glasses of water a day. During the summer, whenever I go out to places like the playground or roller-skating, I bring a backpack, and I always make sure to have water with me”*

(child, Lublin)

*“My older brother drinks as much as two liters of water per day, while I usually consume just half a liter”*

(child, Rosko)

### 3.6. Physical Activity and Leisure Free Time of Primary School Students

Primary school students participating in the research like sports and physical activity. It is noticeable that they eagerly attend extracurricular activities such as gymnastics, horseback riding, swimming, football, judo, and hockey. Engaging in these activities brings them pleasure, a sense of satisfaction, and pride in their accomplishments and exercises. In the entire research, only a few children, typically overweight boys, did not enjoy physical exertion.

In their free time, students primarily engage in play. They also enjoy spending time with peers, such as riding bicycles, rollerblading, or using scooters. The online world plays a significant role in their lives, especially on weekends. During ordinary weekdays, most have parental controls in place, although students from larger cities sometimes use computers and smartphones during the week. Regardless of gender and place of residence, the participants mentioned that they enjoy online games, such as Minecraft, watching funny videos on Tik Tok, YouTube, following the activities of online creators and influencers on Tik Tok, YouTube, and cooking-related shows like ‘Kitchen Revolutions’ and ‘MasterChef Junior’. Students do not typically watch specific programs or channels. The majority claim not to have favorite influencers they follow on social media, and their program choices are random. It is worth noting that children of this age mainly use devices belonging to their parents and do not have their own smartphones or computers yet. Interestingly, the participants show an interest in food-related content, more as a curiosity, like learning how to make blue pancakes. These are usually videos about food rather than healthy eating.

## 4. Discussion

The main meal for all the children taking part in research was dinner. However, not all of the children in the study had breakfast at home, and for many of them, the morning routine was marked by haste, heightened emotions, and a lack of ingrained, internalized practice of having breakfast in the morning.

In people’s diet, particularly in children’s diet, the regular consumption of balanced meals, with a particular emphasis on having a nutritious breakfast, is of paramount importance. Breakfast comes after a long overnight break, during which, the body utilizes a significant portion of its carbohydrate reserves. Furthermore, it occurs before the commencement of the most demanding physical and mental activities. Consuming breakfast has been shown to have a positive impact on cognitive functions [21,22], particularly on the speed of recall and memory. Additionally, it contributes to reduced psychosocial issues [23] and is considered to be a critical factor in preventing overweight, obesity, and other diet-related diseases [24,25]. Moreover, studies have shown that regularly omitting both breakfast and school meals several times a week increases the chances of becoming overweight. Omitting breakfast frequently raises the risk of both central and overall obesity [26].

It is crucial to remember that children and teenagers acquire fundamental knowledge about nutrition primarily at home, by observing the behaviors of other household members and through information intentionally or unintentionally conveyed by their parents [27]. A comprehensive analysis of 57 studies involving 203,706 participants underscored the significant influence of shared family meals on children’s dietary behaviors. Children who take part in meals with other family members tend to consume a higher proportion of healthy foods, resulting in a better overall diet quality. Moreover, these shared meals have been linked to a reduced risk of obesity, an increased frequency of fruit and vegetable consumption, whole grains, and calcium-rich foods. Additionally, family mealtime helps to reduce the possibility of breakfast omission [28]. This time spent together at the dinner table serves as a vital social setting where children can eat with their parents, who are regarded as their primary role models [29]. Encouraging children to share meals, emphasizing regular family breakfasts, and promoting the consumption of dairy products, vegetables, fruits, and healthy snacks, with reasonable restrictions, have all shown positive effects on children’s eating habits [30,31,32].

Combining meals with watching TV or using a tablet is incorrect practice. In this study, a few primary school students consumed their home-cooked meals in this way. Such behaviors encourage overconsumption, disrupt control over what and how we eat (meals/food), and reduce hunger and satiety signals. Research indicates a lower consumption of vegetables, fruits, and grain products, as well as an increased frequency and quantity of highly processed, palatable foods such as fast food, snacks, and sugary drinks among children who engage in eating while watching TV, using a computer, and a phone, etc. [33,34].

Children’s knowledge about food, their preferences, and finally their eating behaviors are closely linked to their parents’ food preferences, beliefs, and attitudes. This is a result of exposure and easy access to the products that parents like, purchase, and consume. Parental beliefs about which foods are healthy and their own dietary experiences also influence children’s food choices [35]. Therefore, the role of parents in creating healthy eating habits is crucial, as it was confirmed in this study.

During the school-age period, food preferences and eating habits are formed, emphasizing the need for a diverse selection of foods in children’s meals and encouraging them to try new or less familiar dishes [4,36,37].

It is essential to introduce a wide variety of foods into children’s meals and encourage them to explore new or less familiar dishes during the school-age years when food preferences and eating habits are being established [4,36,37]. In this study, among students aged 7–9, there were those classified as “reluctant”, and their diets were limited and characterized by a small selection of products. These students were very unwilling to consume new, less familiar products or dishes they disliked. A common phenomenon observed among early school-age children is picky eating, which involves a tendency to reject a wide range of food products, including familiar and previously consumed food, as well as food regularly consumed by other family members [38,39]. Another concerning phenomenon is food neophobia, defined as an attitude towards eating characterized by a persistent aversion to consuming new foods, avoiding the tasting of unfamiliar products, and reluctance to accept new flavors or unfamiliar food textures. Food neophobia is a significant issue from both psychological and dietary perspectives. Given that it occurs during early childhood, it can significantly influence a child’s dietary choices, shape taste preferences, and impact the quality of a child’s diet [40]. Therefore, early recognition this problem becomes crucial in creating the later dietary behaviors of students.

It is also important to remember that children’s food preferences change through experiences. Therefore, it is crucial to allow them to have direct contact with various foods. During the school-age period, children become more aware of their food preferences through tasting, smelling, touching, and visual examination of food items. This awareness results from observing their environment, including family, children at the same age and teachers. It is a time when children start making their first conscious decisions about food choices and creating their food preferences. These early eating behaviors can significantly impact their future health, with unfavorable behaviors often being linked to adolescent and adult health issues such as obesity and non-communicable diseases [41,42].

Exposure to new food products has a cumulative effect. The more new products and dishes appear in a child’s diet, the quicker they gain acceptance [42]. When food is readily available and prepared for consumption, children are more likely to reach for it and consume it. A higher consumption of fruits and vegetables has been observed when these items are placed in easily accessible locations for children, often in convenient, child-sized portions (e.g., sliced apples, carrot sticks, and cherry tomatoes) [35]. Another technique involves combining the pleasant taste of sweetness with less acceptable flavors. Multisensory exposure is also essential, engaging different senses such as taste, smell, touch, and sight in getting to know new food items. This significantly increases the consumption of foods that children and adolescents may initially find unappealing [41,43].

Numerous studies [44,45] indicate that dietary patterns among children and youth do not meet the recommended guidelines, including insufficient consumption of vegetables and fruits and excessive intake of high-fat, high-sugar, and high-salt foods. Data from a study on the dietary behaviors and nutritional status of the Polish population conducted in 2019–2020 [46] confirmed that only a small percentage of children reported the daily consumption of vegetables and fruits, and even fewer students reported eating them multiple times a day. Similar results were obtained in the present study, where 7–9-year-old students lacked knowledge about the recommended intake of fruits and vegetables, highlighting the importance of interventions in schools and community programs aimed at increasing the consumption of vegetables and fruits. These interventions are crucial for promoting and making healthy dietary choices to maintain good physical and mental health and prevent various diseases [47,48]. In this study, it was shown that a key indicator of healthy eating among 7–9-year-old students is the consumption of primarily vegetables and fruits. Students also accurately recognized items that are indicative of an ‘unhealthy’ diet, such as sweets, salty snacks, and fast food products, which suggests a persistent lack of change in this respect over the past two decades [49].

Similar results were also reported by other authors, noting a good nutritional knowledge of primary school students despite poor eating habits. The authors showed that adoption of healthy eating practices is difficult due to food preferences, family factors (parental style and knowledge), food availability, time constraints, and convenience [50].

Consuming sweets, fast food, sugary beverages, and adding extra salt or sugar to drinks and meals can lead to the development of unfavorable taste preferences in adults [3,4]. Children participating in this study were aware of the need to limit such products. They correctly identified “healthy” and “unhealthy” foods. It is also worth noting that sedentary behaviors (such as watching TV, using computers, and reading books or magazines) and low physical activity influence the increased consumption of these products [51,52,53,54]. In this study, the profile of students with low physical activity was described as “indifferent” in terms of dietary behavior, and their diet was characterized by numerous nutritional mistakes. What is interesting is that these students tend to reach for undesirable products when spending time with family, out of boredom, during family outings, and at school. Adults often offer children these kind of products.

Water should be the first choice of beverage for children and adolescents, with other drinks being selected in subsequent order due to their sugar content and additives, and their impact on the physical and mental well-being and health of students [55,56,57]. Students (children) participating in the study knew about the importance of daily water consumption and the need to limit other beverages such as juices or sweetened drinks. It is important to emphasize that, in this case, numerous campaigns promoting water consumption have been effective, and the trend of carrying a bottle with a filter and filling it with tap water, as well as the promotion of water stations, has had a positive impact.

Children’s eating and physical activity behaviors are influenced by both individual factors (such as genetics, age, and gender) and environmental factors (including family, children in the same age, community, and society). The various components of lifestyle interact to impact human health and can be interconnected, magnifying their effects. Physical activity is a significant component of a healthy lifestyle [58], which the students in this study, except those who were overweight, engaged in willingly. At the same time, screen time did not appear to be a significant issue, perhaps due to limited computer and smartphone use, as parents enforced parental controls and children did not have their own electronic devices. Reducing sedentary behaviors and increasing physical activity are recommended for this age group. The period between the ages of 6 and 11 is considered to be a crucial time for interventions to boost physical activity [59]. Creating specific motor skills and behaviors in kindergarten and early school-age children is the responsibility of parents with the support of schools [34,60]. Problems with physical activity and excessive computer use among students, especially during the COVID-19 pandemic, were also noted by other authors [61].

Based on the obtained results, it can be concluded that, during the early school years, parents play a significant role in controlling children’s food consumption and dietary choices. Parents also create children’s eating behaviors, often for the rest of their life. Educational efforts initiated from a very young age should aim to instill and maintain a healthy lifestyle, particularly in terms of various activities, with a strong focus on nutrition and physical activity. Understanding the psychoeducational factors that influence children’s eating behaviors and providing proper nutrition education to both children and their parents/guardians, as well as teachers, can support the development of health-promoting dietary behaviors [41]. It is crucial that the effectiveness of nutrition education can be enhanced by combining knowledge with practical sessions, involving parents, guardians, and the school environment, making it a significant element in creating proper dietary behaviors for children [48,62].

Qualitative research allowed obtaining an in-depth picture of the issues, which are sometimes difficult to grasp, as pointed out by other authors [63,64]. FGI revealed what content, from the students’ perspective, should be included in planned nutritional programs. This is a good example of taking into account the “student voice” in planning educational strategies. Our results indicate the need to educate children in conjunction with the education of their parents and even entire families. According to other authors, two-way communication between home and school is crucial for the successful implementation of this intervention [65].

## 5. Limitation

The conducted study has several limitations that are worth noting. Some of these limitations are inherent to qualitative research, which, due to its small sample sizes, may not be representative. However, when combined with the results of quantitative research, as is the case in the “Junior-Edu-Żywienie (JEŻ)” project, a deeper and more comprehensive explanation of the quantitative research findings was provided.

Our results affect students from Poland, which may be influenced by cultural factors. This fact affects the dietary behaviors and eating patterns specific to the given population. Hence, while our findings cannot be generalized to other populations (regions of the world), it can serve as guidance for other researchers and agencies involved in health policy, the development of school programs, and the implementation of educational initiatives.

## 6. Conclusions

Among the analyzed students aged 7–9, based on their engagement with nutrition, the following profiles were identified: “engaged”, “obedient”, “reluctant”, and “indifferent”. Generally, it can be stated that primary school students’ diets are primarily influenced by their families and schools. Children of nutritionally aware parents tend to eat more correctly and display a significant nutritional awareness. Conversely, children of busy parents who lack time for meal preparation more often have poor eating habits and tend to replicate their parents’ dietary mistakes. Parents who struggle to determine what is healthy or not, despite their desire for proper nutrition, introduce this ambivalence into their children’s nutritional awareness. Students of this age are not entirely sure about what is “healthy” or “unhealthy”, although they can correctly categorize specific food products into these groups based on these labels. However, they have a problem matching these categories with nutritional components. They are familiar with terms such as dietary fiber and fats but do not know which foods are sources of these nutrients or the roles they play in the body.

Students are not sure about the number of meals they should have each day, the recommended portion sizes of foods (vegetables and fruits), and whether consuming sweets occasionally still makes their diet appropriate. Students appreciate homemade meals due to their diversity, better alignment with their preferences, and the time when they can enjoy them, often with family. Therefore, they view the pandemic period positively, during which, they spent more time with their families and could prepare meals together, even though they missed physical activity and the company of children in their age. In conclusion, it can be stated that nutrition education, using credible and reliable educational materials targeted at all the influential environments related to students’ dietary attitudes, especially parents and teachers, as well as students, can yield the desired results for all three components of children’s food and nutrition attitudes. It can also help to create their dietary choices, which are the essence of nutrition and health education.

## Figures and Tables

**Table 1:** Typology of primary school students based on their level of involvement and awareness of nutrition.

Criteria	Primary School Students Profile			
	“Engaged” *n* = 15	“Obedient” *n* = 47	“Reluctant” *n* = 10	“Indifferent” *n* = 6
Involvement in nutrition-related issues	High level	Medium level	Medium level	Low level
Approach to nutrition	They eat healthy, following dietary rules, usually at homes	They eat healthily and rationally because their parents require it of them; if they could, they would chose junk food more often	They eat healthily, do not exclude valuable products, like vegetables and fruits, but it is a selective choice, quite monotonous, and not very diverse	They do not care about what, when, how much, and how they eat; they often have excess weight, spend little time outdoors, and lead a sedentary lifestyle
Parental responsibility for imparting knowledge and instilling healthy behaviors in children	High	High	High	Low
Parental restraint regarding the subject of nutrition	High level	Medium level	High level	Low level
Knowledge about proper nutrition	High level	Medium level	Medium level	Low level

**Table 2:** Linking primary school students’ emotions with the type of meals they choose.

The Type of Emotions	The Type of Meal	Frequency of Occurrence
Sadness, the desire for comfort	Sweets, salty snacks	Often
Irritation, anger	Sweets, fruits	Seldom
Boredom	Easy divisible products (seeds, fruits), snacks, sweets	Often
Happiness, a desire for reward	Sweets, fast food	Seldom

**Table 3:** Reasons for not consuming breakfast among primary school students aged 7–9 years.

Reason	Description	Gender	Place of Residence
Haste	A short amount of time before leaving home, lack of routine, and the need to wake up earlier to have a meal	Boys and girls	Frequently, it refers to larger cities, where students commute to school, sometimes even for several minutes
Appetite loss	The effect of stress, called ‘tight stomach’ (more common in the first grades) and a supper that is too abundant and/or eaten too late	Boys and girls	Applies to every type of location
Lack of routine	Breakfast is not a priority in this group, or an established behavior; going to school is often rushed, sometimes with negative emotions	Boys more often	Slightly more common in larger and medium-sized cities

**Table 4:** ‘’Healthy’’ food products, opportunities and frequency of consumption among primary school students.

Products	Perception	Frequency of Consumption	Opportunities for Consumption
Fruits	Preferred, especially sweet such as bananas, grapes, apples	The vast majority consume daily and only a few consume a few times a week	Very often as a snack at home or at school
Vegetables	Rather preferred; students know that they should consume them and it is not a choice but a healthy behavior started by their parents	The majority daily, a small group a few times a week, and a few individuals less than once a week	Majority of students eat vegetables only for lunch, often in sandwiches, rarely as snacks (only a few students) and in other meals
Grain products	In the students’ understanding, it mainly refers to bread (both white and whole-grain bread); very limited knowledge about fiber and its role in the functioning of the human digestive system	Daily bread, less often grains, rice, most consume them roughly once a week, with a few individuals consuming them less frequently. Some of the respondents have oatmeal for breakfast every day	A significant majority consume bread with practically every meal
Milk and dairy products	Preferred by the majority of children; the main mentioned nutrient is protein, and some also mentioned calcium, which strengthens bones and teeth	The vast majority consume daily	Breakfast, second breakfast, snack, supper
Pumpkin seeds, sunflower seeds, nuts such as walnuts, almonds	It is a form of snack for a significant number of respondents; limited knowledge about impact on the human body	A large portion of respondents consume them several times a week, with a few individuals consuming them once a month or less. Students from small towns and villages only consume sunflower seeds in the summer when there is a season for harvesting from their own gardens	For the vast majority, snacks are a time-filler, a boredom buster
Fish and fish products	Not everyone likes them, but parents and teachers recommend their frequent consumption; most of the surveyed individuals perceive fish as healthy because they contain protein and vitamins	A lot of children consume once a week (on Fridays), while some of them do it less frequently (even only once a year on special occasions or holidays)	Mainly for lunch, for supper—smoked fish and for sandwiches—canned fish

**Table 5:** “Unhealthy” food products, opportunities, and the frequency of their consumption by students.

Context	Type of Products	Description	Gender and Place of Residence
Family	Sweets, sweetened drinks, salty snacks, fast food	During a family event, e.g., a birthday, or spending time together, e.g., watching a match, game night, social meeting; sometimes, fast food replaces dinner when parents do not have time to prepare it, several times a month or less often	Boys and girls, regardless of the type of location, in the case of fast food, in larger and medium-sized cities, there is ordering takeout food
Social	Sweets, sweetened drinks, salty snacks	In company, while playing and spending time together, parents most often give their children this type of products	Boys and girls regardless of the type of location
Individual	Sweets, salty snacks	At home, of boredom, as a time-filler, and at school, during breaks when it is possible to make purchases from vending machines or the school store	Boys and girls regardless of the type of location
Travel	Fast food	On school trip On family trip	Boys and girls regardless of the type of location

## Data Availability

Data are contained within the article and Supplementary Materials.

## Supplementary Materials

The following supporting information can be downloaded at: https://www.mdpi.com/article/10.3390/nu15224732/s1, supplementary file: Focus Group Interview scenario.
