# Supplementary material for: The Attitudes of 7–9 Year Old Primary School Students towards Food and Nutrition: Insights from Qualitative FGI Research—The Junior-Edu-Żywienie (JEŻ) Project
Source: Nutrients. 2023 Nov 9;15(22):4732. doi: 10.3390/nu15224732 (PMC10674932; doi:10.3390/nu15224732)
Supplement: Supplementary file 1 [file nutrients-15-04732-s001.zip › nutrients-2698772-supplementary.pdf]

"FOCUS GROUP INTERVIEW SCRIPT AMONG PRIMARY SCHOOL CHILDREN AGED 7-10 YEARS OLD"

1. **Participants of the interview:** 6-8 children aged 7-10 years old

FGI duration: 90 minut

Introduction to the group discussion (5 minutes)

- Moderator's introduction
- Presentation of the research purpose and the purpose of conducting the group interview
- Explanation of the rules for conducting the group interview
- Explanation that the focus group interview will be recorded, and the recorded content is confidential and will be used for research purposes
- Asking participants if they have any questions - moderator's responses and clarification of any doubts
- Informing about the duration of the meeting: up to 1.5 hours
- Brief introduction of meeting participants: child's age, interests/hobbies, etc.

2. **Evaluating children's approach to nutrition (5 – 10 minutes)**

- Who likes eating? Raise your hand!
  - Why do you like/ do not like eating?
  - What do you like to eat the most? List all the foods you like.
  - What you do not like to eat? List all the foods you do not like.

The moderator can ask the following questions in each topic:

- Why yes / no?
- What is the reason for this?

- In your opinion do you eat healthily?
  - Why do you think so? What factors contribute to it?
  - What kind of foods do you eat that indicate you are eating healthily / not healthily?
  - In your opinion, who eats healthily?
    - Why and what kind of products do they consume?
    - What does such a person avoid?
- When do you usually eat your meals?
  - When are you hungry?
  - When you are feeling sad?
  - When you are feeling anxious?
  - When you want to comfort yourself?
  - When you want to reward yourself?
  - When you are bored?
  - When you are in group?

3.**Children's lifestyle regarding nutrition (10 – 15 minutes)**

- How many meals do you typically eat during a day? List them all. Let's try to count them:
  - Which meal do you like to eat the most??
  - Which meal do you think is the most important in your opinion? Why do you think so?
  - Which meal is less important?
  - Do you think it's possible to skip a meal? Why? What do you think will happen? Which meals do you eat at home, and which ones do you and which at school?
- Why do you think we eat breakfast??

„Przeprowadzenie badań naukowych z zakresu żywienia dzieci i młodzieży oraz opracowanie i wdrożenie programu edukacji żywieniowej uczniów klas I-VI szkół podstawowych. Akronim: Junior – Edu-Żywienie (JEŻ)"
Zadanie finansowane ze środków Ministerstwa Edukacji i Nauki na podstawie Umowy nr MEiN/2022/DPI/96 z dnia 07.03.2022 r.

- Do you like to eat breakfast?
- How often do you eat breakfast? Every day? Only sometimes?
- Where do you eat breakfast? At home or at school?
- What is the role of this meal?
- Do you believe that eating breakfast before going to school gives you the energy for learning and playing? Why do you think so?
- Summary: Why do we eat/consume various meals at all? What do you think?
  - What is their role? What benefits do we gain from eating?
  - Do you believe that health and strength depend on what you eat?
  - Why eating is so important?
- Do you prefer to eat meals at home or at school? Why?
- Do you enjoy eating with others or alone?
- What does eating meals at home typically look like?
  - Does the whole family gather, or do you eat alone? What factors influence this?
  - How often do you have meals together? Are there any differences between a regular weekday and a day off or a holiday? What are the differences?

4.Perception of various food groups - recommended food products (10-15 minutes)

- Are there any food groups that parents ask you eat every day, even if you don't like them?
- What are those products? Let's list all of them.

The moderator is trying to include the following products in this category:

- **fish:**
  - How often should we eat fish?
  - How many times a week do you eat fish?
  - Do you understand why fish is considered healthy? What do they contain a lot of?
  - Are you familiar with the concept of "healthy fats"? What does it mean?
  - In what other products can you find these "healthy fats"?
  - What are their effects and functions in our body?
  - Do you know that they affect immunity and memory?
- **fruits and vegetables:**
  - What are your favorite fruits? List them.
  - How many fruits and vegetables do you eat during a day?
  - Do you eat a fruit or vegetable with every meal?
  - How much space should fruits and vegetables occupy on your plate? (The moderator can show printed diagrams if needed.)
  - Have you heard that it's recommended to eat fruits and vegetables at least 5 times a day? Do you think it's a lot or a little?

Pumpkin seeds, sunflower seeds, nuts such as walnuts, almonds:

Do you like these types of products?

- How often do you eat them?
- Do you think they are healthy for the body? Why? How do they affect us?

- **grain products:**
  - What specific products belong to this group? What can you list? (Moderator can suggest: whole grain bread, oatmeal, grains
  - Which ones do you prefer to eat the most?
  - Why are they healthy for our bodies? What do they contain a lot of?
  - Have you ever heard of fiber? What is it? What does it impact?

„Przeprowadzenie badań naukowych z zakresu żywienia dzieci i młodzieży oraz opracowanie i wdrożenie programu edukacji żywieniowej uczniów klas I-VI szkół podstawowych. Akronim: Junior – Edu-Żywienie (JEŻ)"
Zadanie finansowane ze środków Ministerstwa Edukacji i Nauki na podstawie Umowy nr MEiN/2022/DPI/96 z dnia 07.03.2022 r.

- <u>**dairy products:**</u>
  - How often do you eat dairy products such as cheese, milk, and yogurt?
  - Do you like dairy products?
  - What do they affect, what functions of our body?
  - Do they contain a lot of calcium? What do we need calcium for in our body?

5. Perception of different food groups - products not recommended for consumption (10-15 minutes)

- Are there any groups of products that you would like to eat more often, but your parents do not allow you?:
  - What products are these? Let's list them all.
    - Do you think that ... can be eaten every day?
    - How often do you eat/get for these products?
    - Why are these products considered unhealthy? What do they contain too much of? (Moderator can suggest: salt and fats)
    - Why should they be limited?
    - What damage do they cause to our body?

Moderator Our goal is to have the following products in this group:

- Sweets (cookies, bars, candies, sweet cereals (chocolate balls, sweet glazed circles)
- Salted snacks (pretzels, chips, breaded peanuts)
- Fast food (pizza, hamburgers, kebabs, french fries, baguette sandwich)

6. Consuming liquids during the day (5-10 minutes)

- How much liquid/drinks do children like you should consume during the day? What do you think? (You can suggest: 1-1.5 liters, about 2 liters, over 3 liters).
- What drinks do you like? List all types.
- Which of them are healthy, and which are not?

Moderator: "Our aim is to establish a category of sweetened drinks, vegetable-fruit juices, and water:

- Why did you classify as healthy/unhealthy drinks? What's the reason?
- What ingredients do these drinks contain that make them healthy/unhealthy? (You can suggest: sugar in the ingredients)
- How often do you consume drinks from this group? Every day? Once a week? Seldom?

7.Exercise, sport and physical activity (5 – 10 minutes)

- What do you most often do after school in your free time?
  - Do you engage in any sports? Which ones? (Moderator, make sure we're talking about activities outside of physical education classes) How often? How many hours per day/week?
  - Do you enjoy sports activities and physical exercise? How do you feel after physical exertion? What emotions accompany you?
  - Do you believe that being physically active every day is important? Why?
  - Is sports important in your life? Why, what does it give you?
- If someone doesn't like physical activity and sports, what's the reason?
- What do you prefer to do in your free time instead of engaging in sports?
  - Do you even like to listen/watch/read about food and food products?
  - What do you find enjoyable about it? What interests you the most? Why?
  - What isn't enjoyable for you? Is there anything that bores you? Why?
- Do you help with cooking at home?:
  - What do you usually do? What tasks do you perform?

„Przeprowadzenie badań naukowych z zakresu żywienia dzieci i młodzieży oraz opracowanie i wdrożenie programu edukacji żywieniowej uczniów klas I-VI szkół podstawowych. Akronim: Junior – Edu-Żywienie (JEŻ)"
Zadanie finansowane ze środków Ministerstwa Edukacji i Nauki na podstawie Umowy nr MEiN/2022/DPI/96 z dnia 07.03.2022 r.

- What do you like to cook?
- Can you prepare any dishes by yourselves? Which ones?

8. The pandemic and dietary habits (5 minutes)

- Do you still remember the times of the pandemic? You couldn't go to school, and education was remote, so you spent most of your time at home
  - How did your daily routine change during that time? What changes occurred?"
  - How did you feel?
  - How did you spend your time?
  - Did you engage in any additional activities? Did you have more or less physical activity?
  - Do you feel that you ate different foods or meals than usual during that time? What foods do you associate with that period?
  - What types of foods did you eat more?
  - Do you still eat those products? Why yes/no?
  - What types of foods did you eat seldom?
  - Do you feel that you are eating in the same way as during the pandemic?

9. Summary of the group discussion (5 minutes)

- A brief summary of the discussion.
- Is there anything else anyone would like to add?
- Thank you for participating

„Przeprowadzenie badań naukowych z zakresu żywienia dzieci i młodzieży oraz opracowanie i wdrożenie programu edukacji żywieniowej uczniów klas I-VI szkół podstawowych. Akronim: Junior – Edu-Żywienie (JEŻ)”
Zadanie finansowane ze środków Ministerstwa Edukacji i Nauki na podstawie Umowy nr MEiN/2022/DPI/96 z dnia 07.03.2022 r.
